# Supplementary material for: The Evolution of Highly Pathogenic Avian Influenza A (H5) in Poultry in Nigeria, 2021–2022
Source: Viruses. 2023 Jun 17;15(6):1387. doi: 10.3390/v15061387 (PMC10301279; doi:10.3390/v15061387)
Supplement: Supplementary file 1 [file viruses-15-01387-s001.zip › viruses-2420536-supplementary.pdf]

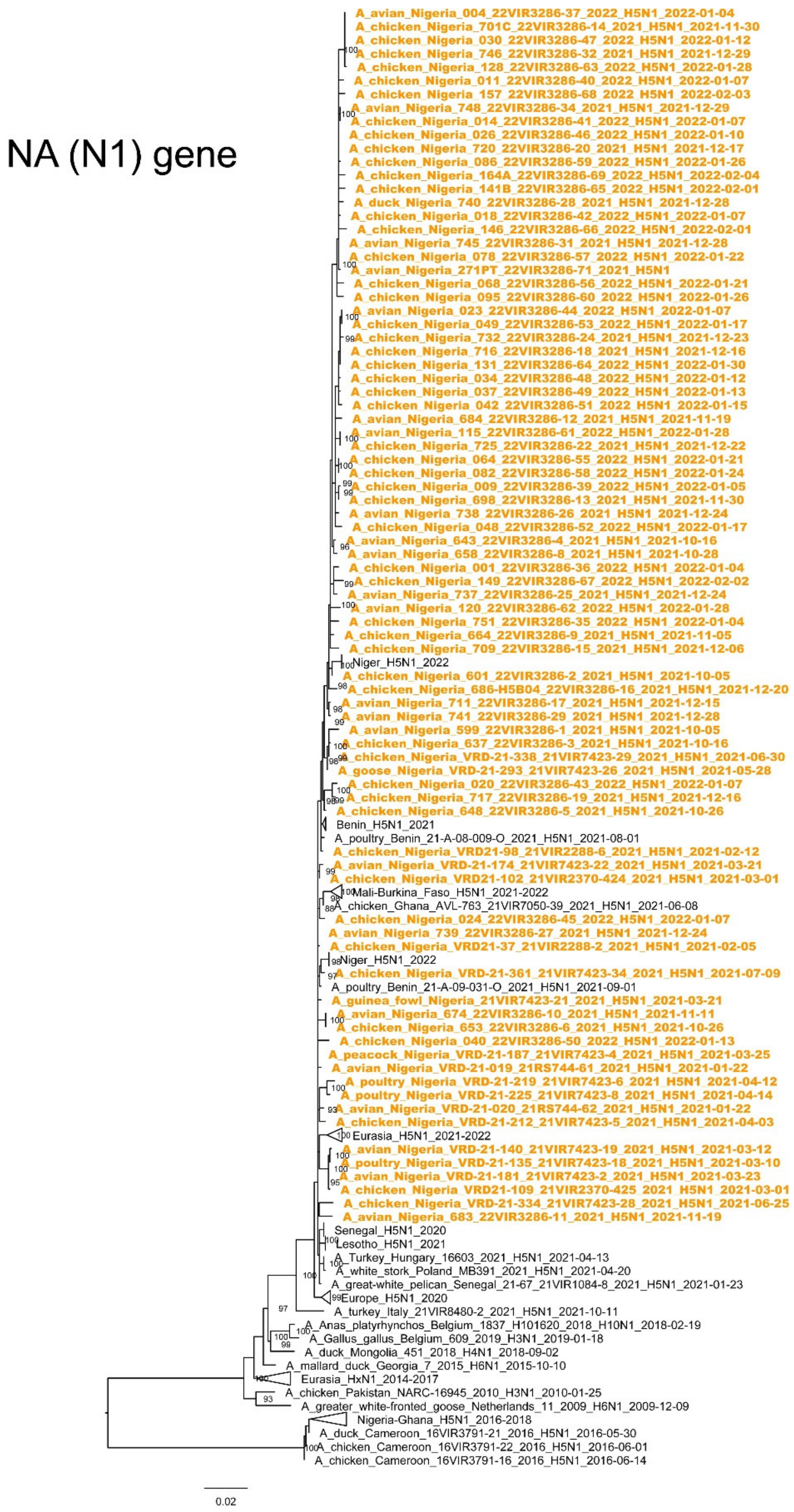

Figure S1. Phylogenetic tree of the nucleotide sequence of the NA (N1) gene obtained with the Maximum Likelihood method by using IQ-TREE v.1.6.12. The H5N1 Nigerian viruses from 2021 are shown in orange. The Ultra-fast bootstrap values higher than 80% are indicated near the nodes.

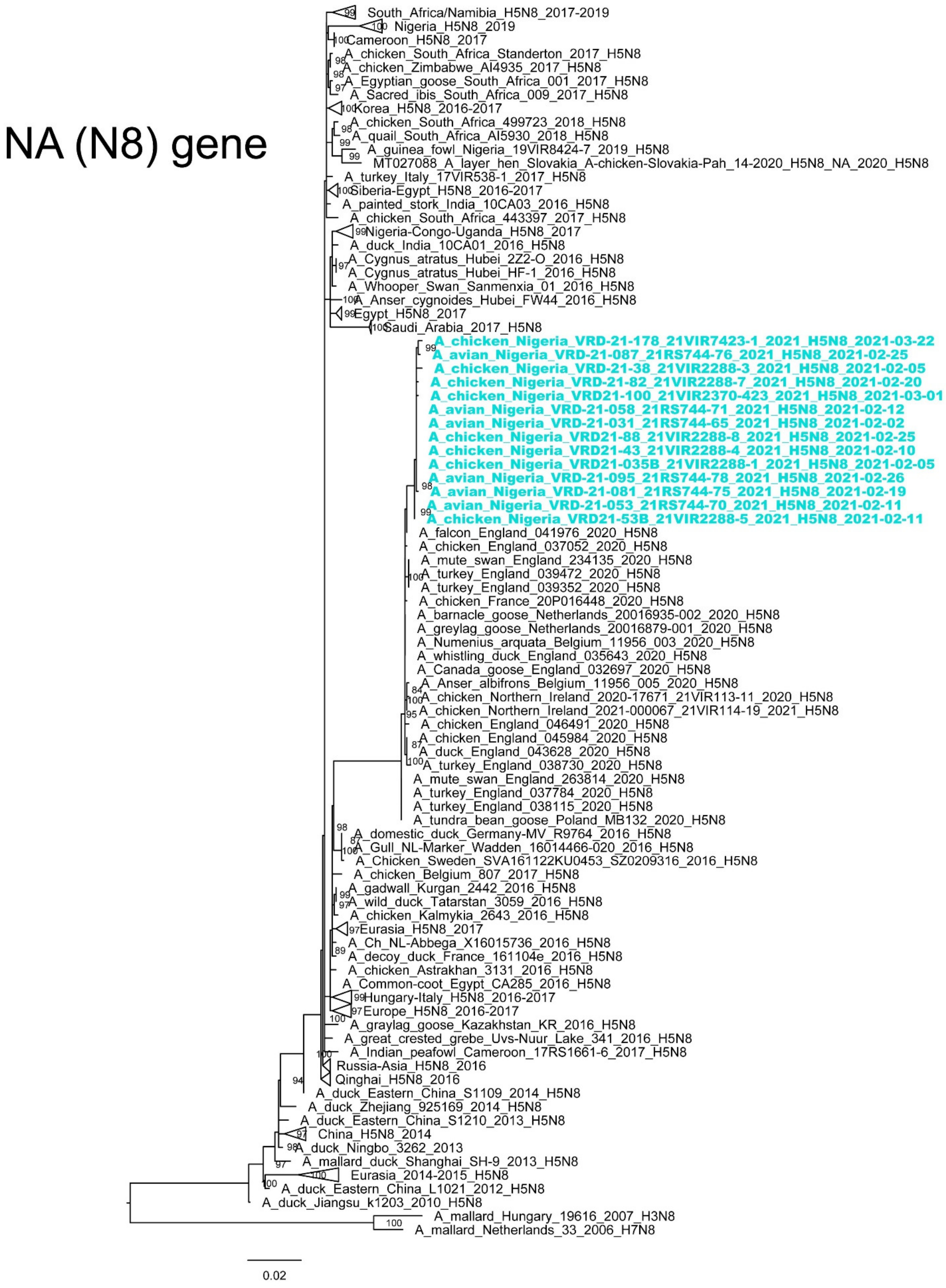

Figure S2. Phylogenetic tree of the nucleotide sequence of the NA (N8) gene obtained with the Maximum Likelihood method by using IQ-TREE v.1.6.12. The H5N8 Nigerian viruses are shown in light blue. The Ultra-fast bootstrap values higher than 80% are indicated near the nodes.

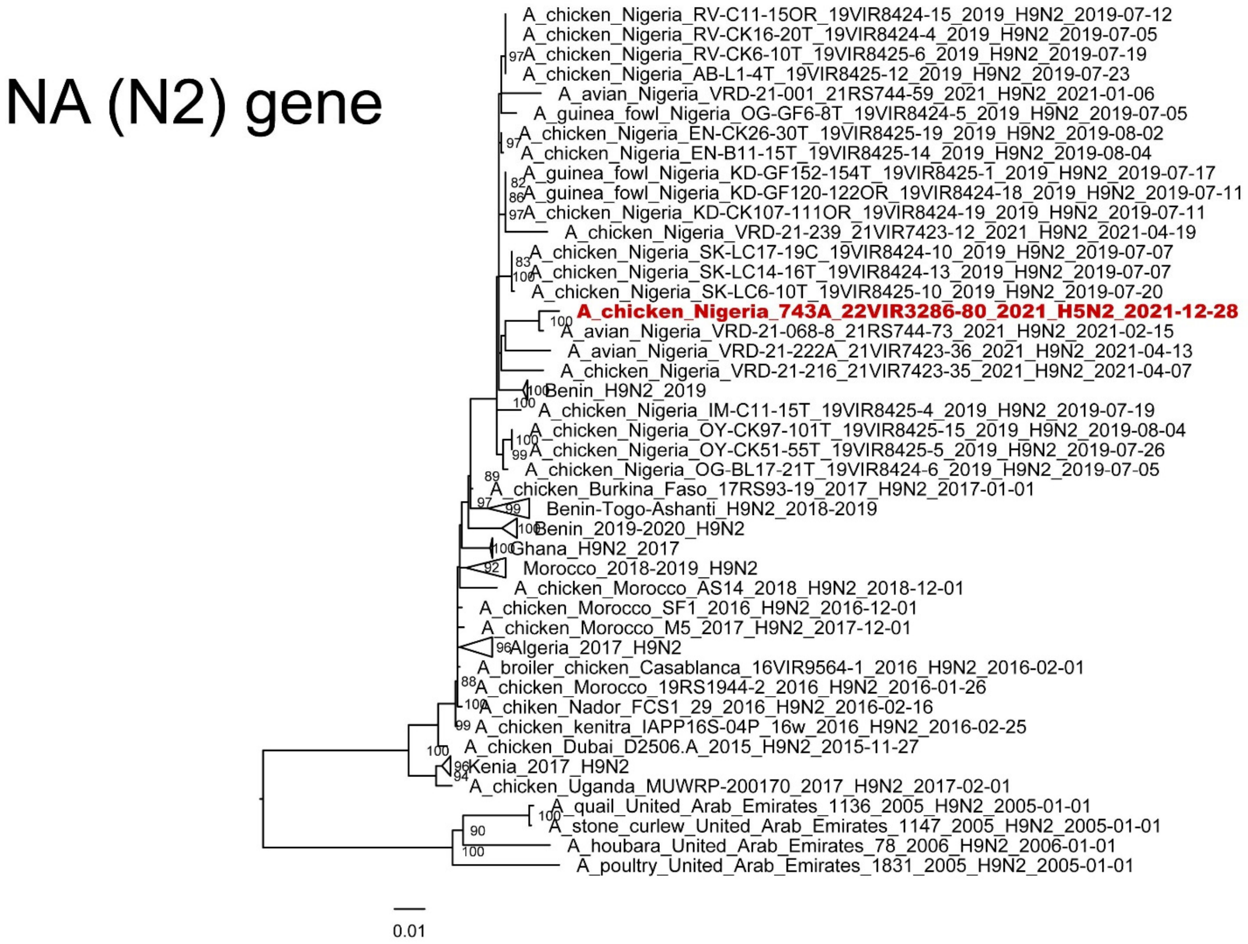

Figure S3. Phylogenetic tree of the nucleotide sequence of the NA (N2) gene obtained with the Maximum Likelihood method by using IQ-TREE v.1.6.12. The H5N2 Nigerian virus is shown in red. The Ultra-fast bootstrap values higher than 80% are indicated near the nodes.

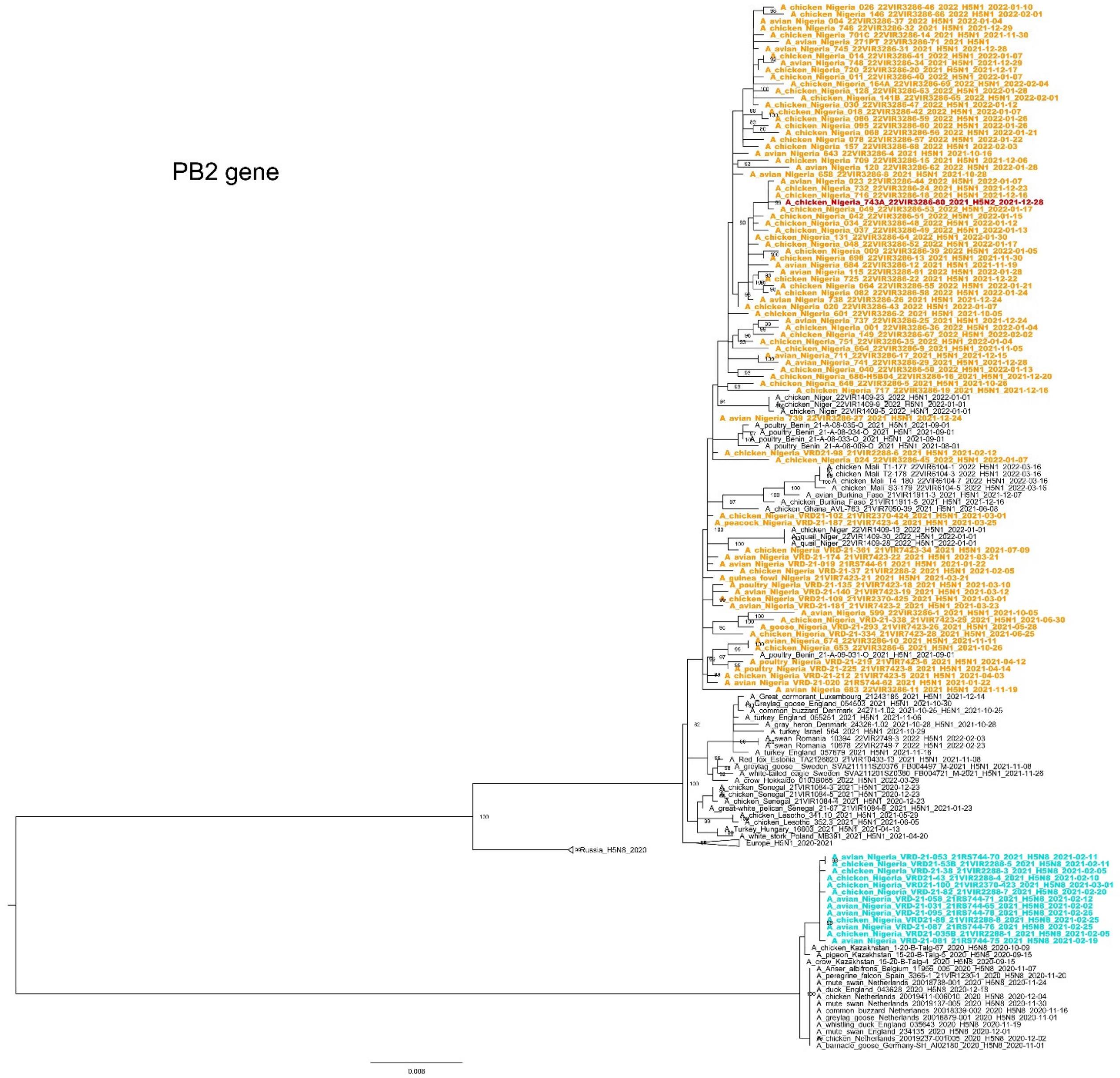

Figure S4. Phylogenetic tree of the nucleotide sequence of the PB2 gene obtained with the Maximum Likelihood method by using IQ-TREE v.1.6.12. The H5N1 Nigerian viruses from 2021 are shown in orange, H5N2 reassortant virus in red, and H5N8 in light blue. The Ultra-fast bootstrap values higher than 80% are indicated near the nodes.

PB1 gene

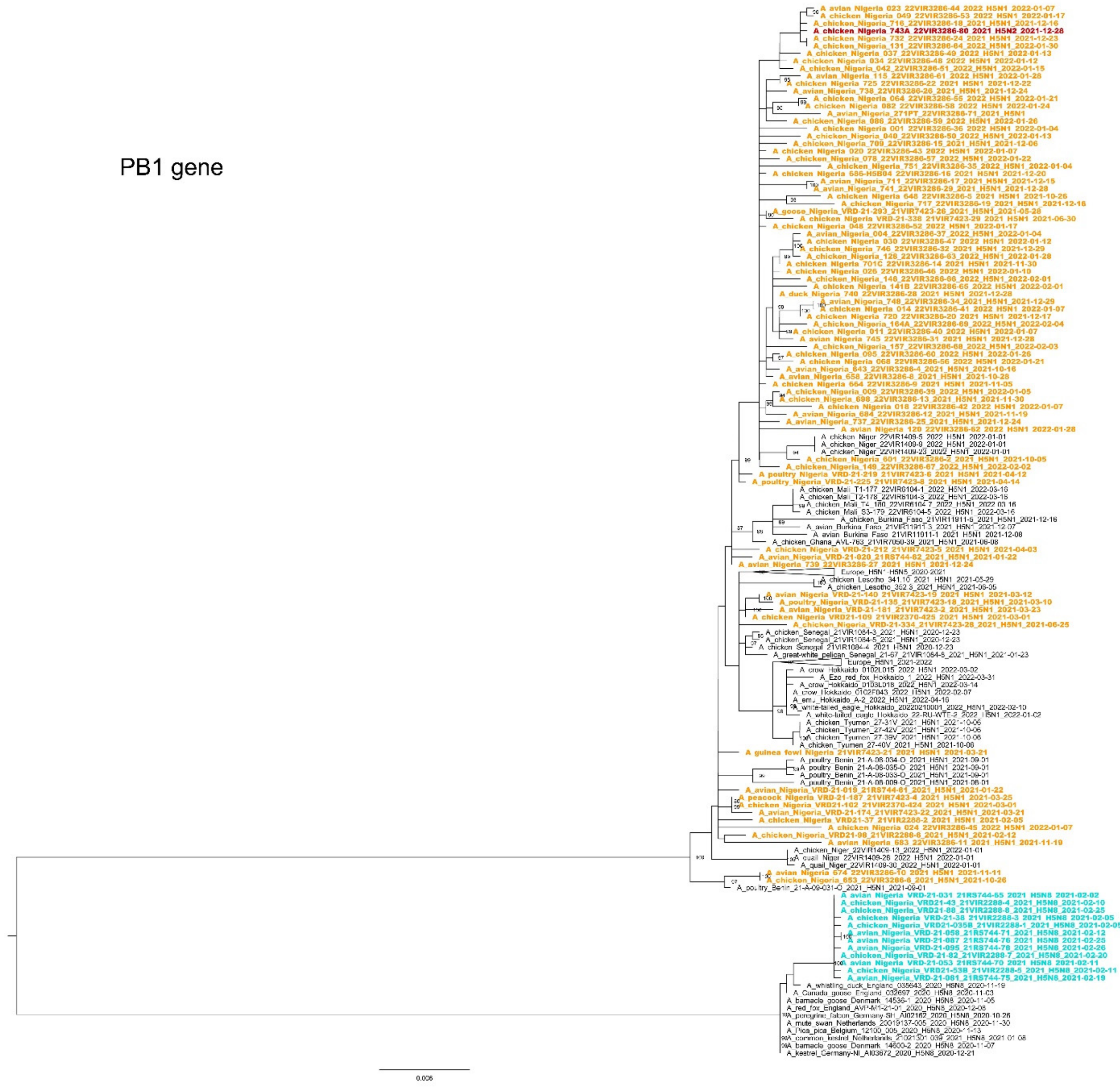

Figure S5. Phylogenetic tree of the nucleotide sequence of the PB1 gene obtained with the Maximum Likelihood method by using IQ-TREE v.1.6.12. The H5N1 Nigerian viruses from 2021 are shown in orange, H5N2 reassortant virus in red, and H5N8 in light blue. The Ultra-fast bootstrap values higher than 80% are indicated near the nodes.

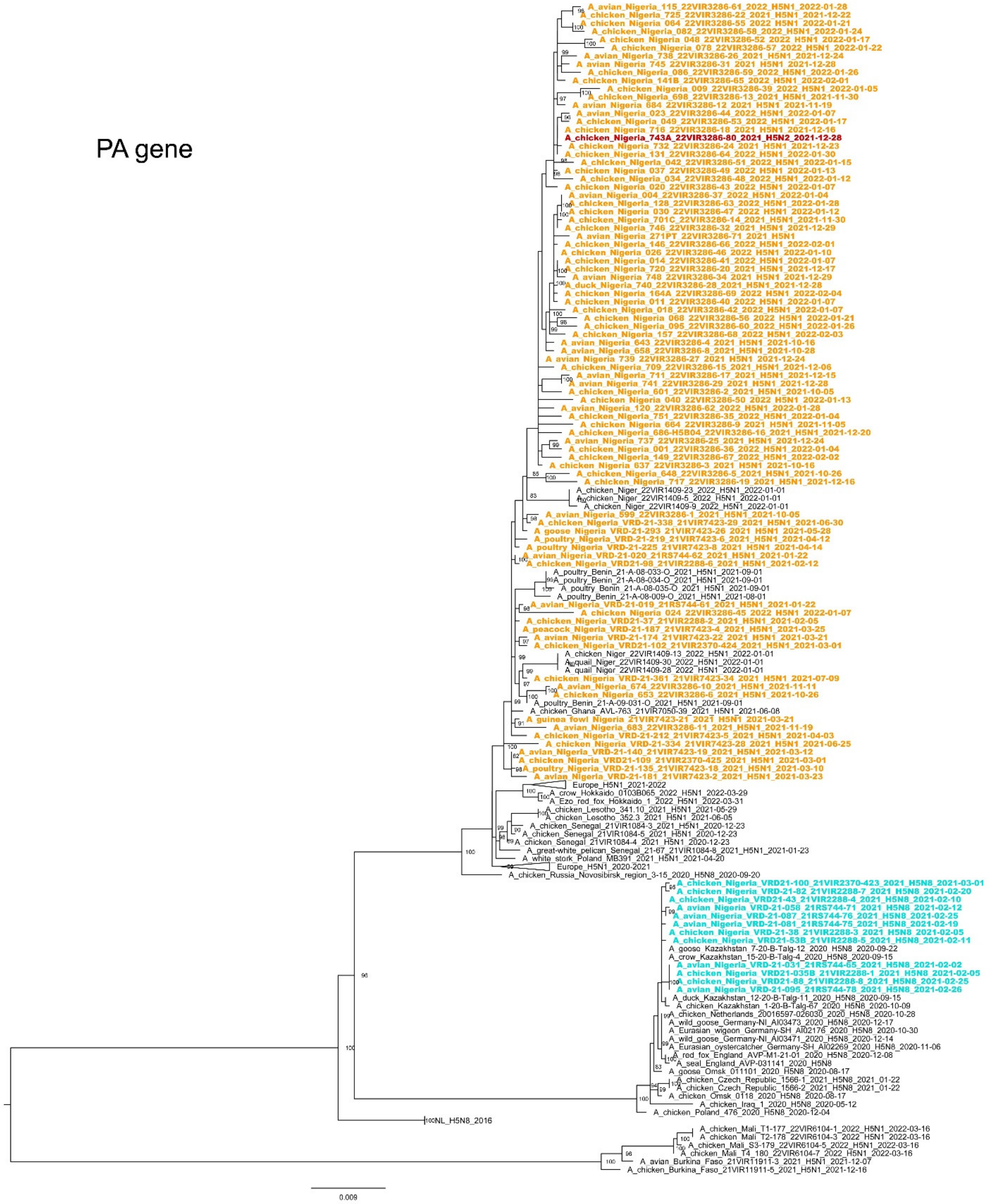

Figure S6. Phylogenetic tree of the nucleotide sequence of the PA gene obtained with the Maximum Likelihood method by using IQ-TREE v.1.6.12. The H5N1 Nigerian viruses from 2021 are shown in orange, H5N2 reassortant virus in red, and H5N8 in light blue. The Ultra-fast bootstrap values higher than 80% are indicated near the nodes.

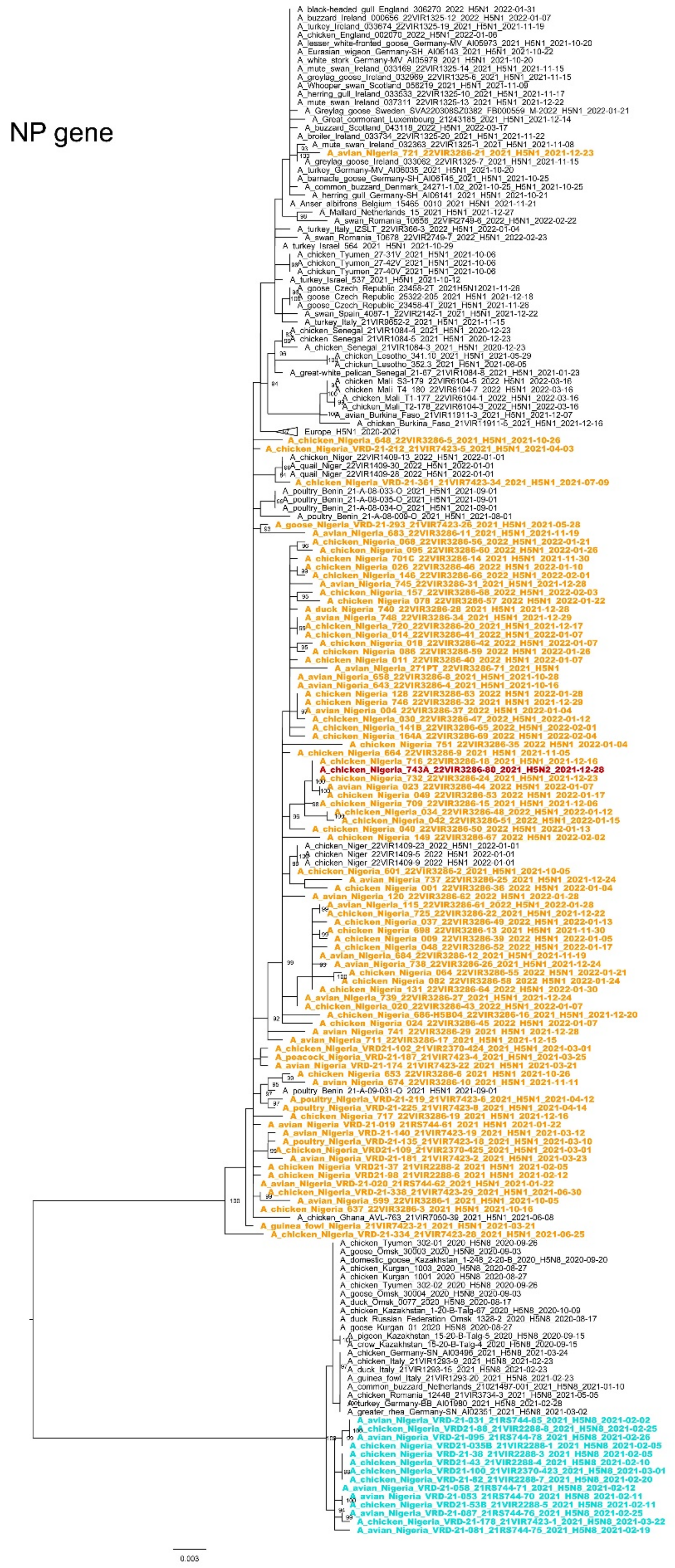

Figure S7. Phylogenetic tree of the nucleotide sequence of the NP gene obtained with the Maximum Likelihood method by using IQ-TREE v.1.6.12. The H5N1 Nigerian viruses from 2021 are shown in orange, H5N2 reassortant virus in red, and H5N8 in light blue. The Ultra-fast bootstrap values higher than 80% are indicated near the nodes.

M gene

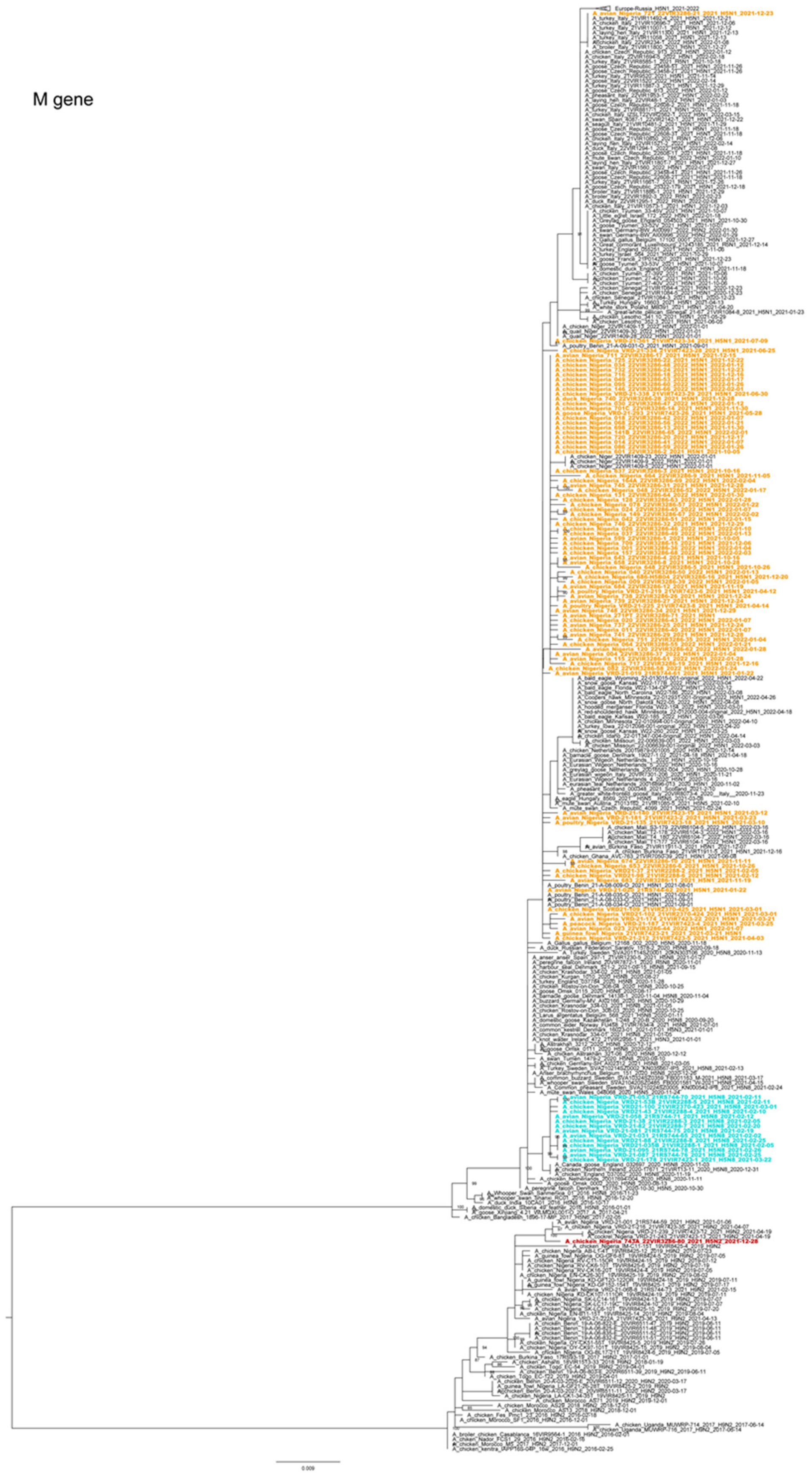

Figure S8. Phylogenetic tree of the nucleotide sequence of the M gene obtained with the Maximum Likelihood method by using IQ-TREE v.1.6.12. The H5N1 Nigerian viruses from 2021 are shown in orange, H5N2 reassortant virus in red, and H5N8 in light blue. The Ultra-fast bootstrap values higher than 80% are indicated near the nodes.

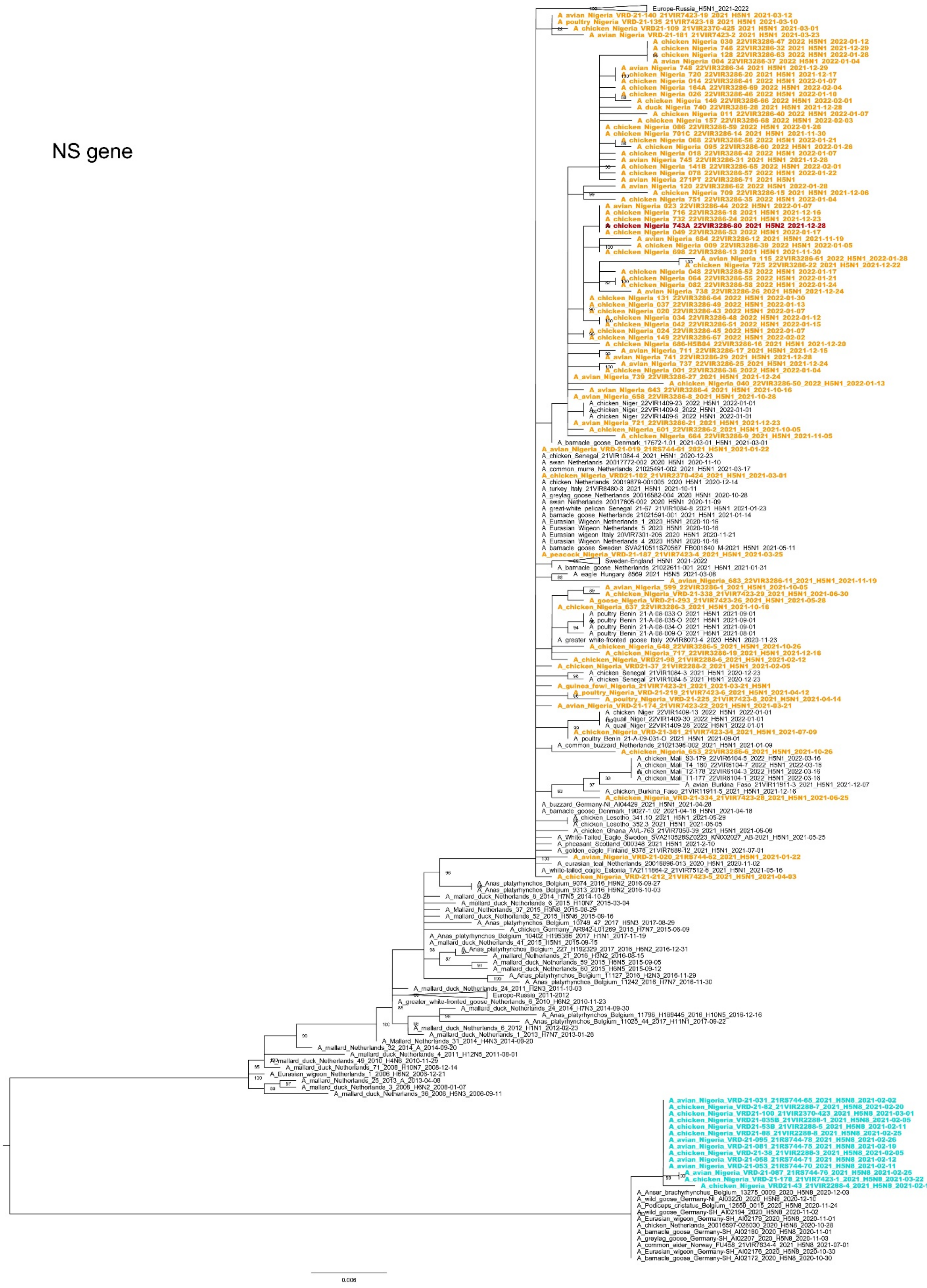

Figure S9. Phylogenetic tree of the nucleotide sequence of the NS gene obtained with the Maximum Likelihood method by using IQ-TREE v.1.6.12. The H5N1 Nigerian viruses from 2021 are shown in orange, H5N2 reassortant virus in red, and H5N8 in light blue. The Ultra-fast bootstrap values higher than 80% are indicated near the nodes.

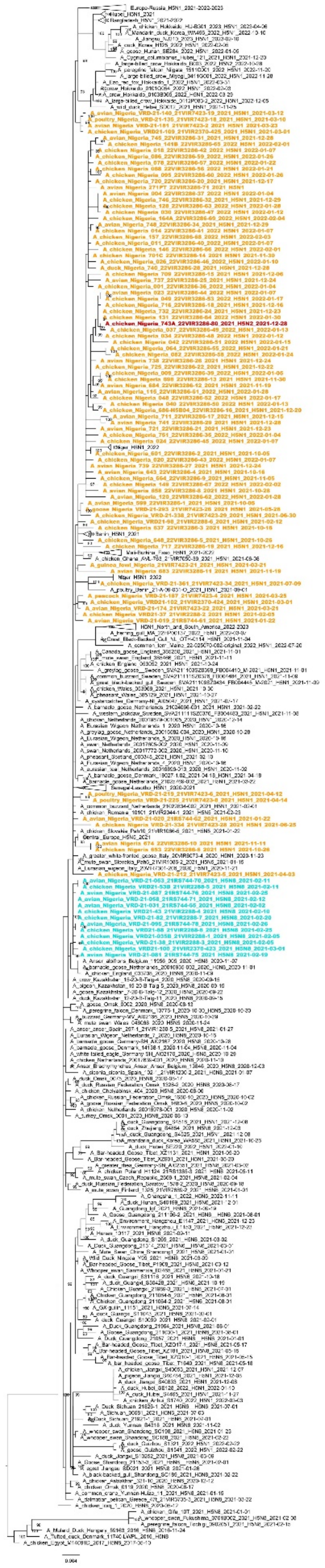

Figure S10. Phylogenetic tree of the nucleotide sequence of the HA gene obtained with the Maximum Likelihood method by using IQ-TREE v.1.6.12. Sequences of representative viruses from Asia, Europe, America have been included in the dataset. The H5N1 Nigerian viruses from 2021 are shown in orange, H5N2 reassortant virus in red, and H5N8 in light blue. The Ultra-fast bootstrap values higher than 80% are indicated near the nodes.
